# Research Enhancing Acidic Mine Wastewater Purification: Innovations in Red Mud–Loess

**DOI:** 10.3390/ma17092050

**Published:** 2024-04-26

**Authors:** Wdah. T. Salih, Zean Xiao, Xiaoqiang Dong

**Affiliations:** College of Civil Engineering, Taiyuan University of Technology, Taiyuan 030024, China; wdahsalih3543@gmail.com (W.T.S.); xiaozean@tyut.edu.cn (Z.X.)

**Keywords:** heavy metal-contaminated soil Cd, solid waste, engineered barrier, purification heavy metal-contaminated soil

## Abstract

This study investigates the adsorption of cadmium (Cd) by red mud–loess mixed materials and assesses the influence of quartz sand content on permeability. Shear tests are conducted using various pore solutions to analyze shear strength parameters. The research validates solidification methods for cadmium-contaminated soils and utilizes SEM-EDS, FTIR, and XRD analysis to elucidate remediation mechanisms. The findings suggest that the quartz sand content crucially affects the permeability of fine-grained red mud–loess mixtures. The optimal proportion of quartz sand is over 80%, significantly enhancing permeability, reaching a coefficient of 6.7 × 10^−4^ cm/s. Insufficient quartz sand content of less than 80% fails to meet the barrier permeability standards, leading to a reduced service life of the engineered barrier. Adsorption tests were conducted using various pore solutions, including distilled water, acidic solutions, and solutions containing Cd, to evaluate the adsorption capacity and shear characteristics of the red mud–loess mixture. Additionally, the study examines the behavior of Cd-loaded red mud–loess mixtures in various pore solutions, revealing strain-hardening trends and alterations in cohesiveness and internal friction angle with increasing Cd concentrations. The analysis of cement–red mud–loess-solidified soil demonstrates enhancements in soil structure and strength over time, attributed to the formation of crystalline structures and mineral formations induced by the curing agent. These findings provide valuable insights into the remediation of cadmium-contaminated soils.

## 1. Introduction

The rapid evolution of global production technology heightens the impact and threat of human activities on water and soil environments. However, these environments are fundamental to human survival, with their health intricately linked to the sustainable development of societies. Consequently, evaluating, controlling, and restoring water and soil environmental pollution, along with efficiently utilizing solid waste resources, have emerged as crucial imperatives in environmental protection, both nationally and globally. Soil heavy-metal contamination has broken worldwide environmental, social, and safety standards. The overall soil environment in China still faces significant challenges. The environmental quality of mining waste-impacted soil is concerning, especially the pollution with heavy metals and organic matter [1,2]. The “National Soil Pollution Survey Bulletin” released in 2014 gave the situation of heavy metal pollution in Chinese soil as exceeding the standard [3]; the over-standard rates of eight inorganic pollutants comprising copper, mercury, arsenic, cadmium, lead, chromium, zinc, and nickel were 2.1%, 1.6%, 2.7%, 7.0%, 1.5%, 1.1%, 0.9%, and 4.8%.

Operators can manually manage and treat acidic mining effluent at a low cost. However, due to regional transitions and coal resource depletion, many mines have been abandoned, resulting in acidic mine effluent from these abandoned coal mines threatening ecosystem safety [4,5]. The acidic mine wastewater from abandoned coal mines has historically not been effectively treated and improved, leading to a significant portion entering groundwater and surface water, causing irreversible damage to the ecological environment [6,7,8,9]; conversely, acid mine wastewater exacerbates water shortages. In South Africa, acidic mine effluent originates from several abandoned mines and tailings in the Witwatersrand Basin [10]. In the abandoned coalfield of Shanxi, China, a substantial amount of acidic mine effluent has accumulated, reaching the underground aquifer through floor fissures, causing permanent groundwater contamination [6]. Highly corrosive acid mine effluent damages 19,300 km of rivers and 720 square kilometers of lakes worldwide, flooding 2260 abandoned mines in Canada [11]. Australia, a significant producer of essential minerals, faces water and land pollution from acid mine effluent. Cleaning it in abandoned mines costs three times more than during operations [4]. Therefore, the contamination of water and soil by acid mine wastewater and the search for appropriate treatment solutions have long been global issues.

The US-EPA proposed permeable reactive barrier (PRB) technology in 1982, and the University of Waterloo extensively studied and developed PRB applications in the 1990s. Its critical benefits as an in situ remediation technology include transferring pollutants via groundwater gradients without external force devices, occupying no ground space, and being cheaper, easier, and less disruptive to the environment than ex situ restoration technology [12,13]. Furthermore, it is sustainable, with a changeable repair filler, allowing the extended repair of the active medium in the PRB wall. The active medium can be renewed for long-term effectiveness in PRB applications, contributing to the efficient removal of contaminants in various in situ treatments. Although PRB may effectively remediate some pollutants, its efficacy is subject to variability based on site-specific factors and the characteristics of the contaminants [14,15]. Thus, PRB technology has evolved into an in situ groundwater remediation technology with great engineering application potential, unlike the high-cost extraction treatment technology. Various methods, including traditional trench installation, caisson installation, continuous excavation, and landfill, are used for PRB in situ remediation projects, depending on the polluted site [15]. Trench installation involves excavating and back to construct a continuous trench and then backfilling the active medium. A caisson-type installation uses prefabricated caissons to aid excavation. When the caissons reach the design depth, the topsoil layer is drained and filled with active media. This method suits polluted areas with large plumes, high pollutant concentrations, and high groundwater flow rates. Continuous excavation and landfill involve machines digging trenches in the planned site and filling them with active medium. Permeable reactive barriers are a very successful in situ technique used for the restoration of aquifers and groundwater [16]. Given the fast movement [17] and restricted natural biodegradation capacity of MTBE [18,19], using PRBs as a means of minimizing or removing the MTBE pollution has great potential. The reactive medium, which is a crucial element of PRBs, is generally chosen based on the characteristics of the pollutants being targeted and the hydrogeological circumstances of the field areas.

In specific cases, PRB has employed a combination of 22% Fe^0^ and 78% concrete as the active medium to remove groundwater trichloroethylene and tetrachlorethylene [20]. The world’s first full-scale reducing PRB, used to treat acidic mine wastewater, removes pollutants and generates alkalinity through sulfate reduction, metal sulfide precipitation, trace element adsorption, and co-precipitation [21,22]. Permeable reactive barriers have existed for well over 15 years. For instance, in one application, a PRB wall employed apatite II as the active medium, effectively removing Pb^2+^, Zn^2+^, and Cd^2+^ from mine water while also neutralizing acidic mine water. This demonstrates the longstanding effectiveness and versatility of PRBs in addressing groundwater contamination issues [23]. Researchers in Western Bulgaria implemented a continuous PRB using biodegradable organic materials, crushed limestone, and ammonium phosphate-saturated zeolite [24]. Pilot tests suggested it effectively treats acidic mine effluent with heavy metals and sulfates. In a Spanish mining region, a PRB was built using calcite, plant compost, sludge, and Fe^0^, resulting in neutral water pH after restoration [25]. Moreover, 95% of heavy metal ions, including Al^3+^, Zn^2+^, and Cu^2+^, were removed, enhancing groundwater quality.

Until now, fixed-bed column experiments have been extensively used to replicate PRBs for many types of pollutants, including heavy metals and dyes [26,27]. These studies have used various adsorbents, such as activated carbon and zeolites [28,29]. Red mud, a waste product generated during alumina production, poses significant challenges for disposal. However, its versatile functionality includes extracting heavy metal ions from wastewater and neutralizing acidic wastewater. This is due to its exceptional properties for heavy metal adsorption and alkalinity [30,31]. Furthermore, loess, which has a strong ability to neutralize acidic solutions, demonstrates a particular effectiveness in eliminating heavy metals [32,33].

The selection of PRB active media is pivotal for assessing PRB’s effectiveness in remediating acidic wastewater-contaminated sites in mining areas. Extensive research by scholars has explored the composition, concentrations, and pollutant ranges in acidic wastewater, advancing the development and application of PRB technology for in situ restoration in mining regions.

Zhu et al. [34] employed sulfate-reducing bacteria to treat acidic wastewater from coal gangue mountains, achieving significant removal rates for SO_4_^2−^, Fe^2+^, Mn^2+^, Pb^2+^, and Zn2% at a pH of 7.0. However, this method is not suitable for acidic mine wastewater with low pH values [35].

Leiva et al. [36] effectively utilized graphene materials modified with zinc oxide nanoparticles to remove Mn^2+^ from acidic mine wastewater. They achieved notable adsorption capacities of 12.60 mg/g and 5.60 mg/g at initial pH values of 4.0 and 5.0, respectively. Additionally, Wang et al. [37] discovered that loess soil with a particle size of 0.02–0.002 mm exhibited the highest adsorption capacity for Cd, reaching 1.80 mg/g. Similarly, Doula et al. [38] employed clinoptilolite to address Mn^2+^-contaminated wastewater, achieving an adsorption capacity of 7.69 mg/g. The research indicates that Mn^2+^ can undergo high-concentration surface precipitation on calcite, enhancing its removal via chemical adsorption [39].

PRB technology has spurred research into utilizing industrial solid waste like red mud, steel slag, and fly ash to combat water pollution. Unlike Fe^0^, iron-containing materials, activated carbon, and zeolite, industrial solid waste offers affordability, accessibility, and treatment efficacy. Utilizing alkaline solid waste like red mud aligns with resource utilization imperatives, particularly for acidic mine wastewater with a low pH and heavy metal ions as the primary pollutants. This article proposes a novel approach—using waste for pollution treatment—by employing red mud and loess as the PRB active media to synergistically cleanse acidic wastewater from mining areas.

This study presents an innovative approach to soil remediation, with a particular focus on treating acidic mine wastewater contaminated with heavy metals like cadmium (Cd). The research introduces a novel active material for engineered barriers by combining red mud and loess in a 7:3 mass ratio, demonstrating robust Cd ion adsorption capabilities and efficient acid-buffering properties. It assesses the influence of quartz sand content on permeability and conducts shear tests with various pore solutions to analyze shear strength parameters. Moreover, this study validates the solidification methods for cadmium-contaminated soils and utilizes advanced analytical techniques such as SEM-EDS, FTIR, and XRD to elucidate remediation mechanisms. The findings indicate that insufficient quartz sand may compromise barrier permeability standards, leading to a reduced service life, while an appropriate content enhances soil structure and strength over time. 

## 2. Materials and Methods

### 2.1. Test Materials

The red mud used in this experiment originated from Shanxi Liulin Aluminum Plant and was obtained as a byproduct of the Bayer process employed in alumina production. Red mud is a powdered substance that appears either gray or red due to its varying iron oxide concentration [40]. The loess used in the experiment was sourced from a construction site located in Dongshan District, Taiyuan City, Shanxi Province. The sample was extracted from a depth of about 4 to 5 m below the surface and was classified as Q4 loess. Table 1 shows the key chemical constituents of red mud and loess. Following air-drying and crushing, the red mud and loess samples were subjected to an additional 24 h of drying in an oven set at 105 °C. Once cooled, the materials were sifted and then stored for future use. Additionally, the liquid plastic limit testing revealed that red mud has a plastic limit of 41.72%, a liquid limit of 52.41%, and a plasticity index of 10.69. In comparison, loess has a plastic limit of 15.84%, a liquid limit of 29.71%, and a plasticity index of 13.87. Simultaneously, it elevates the acidity of acidic wastewater, ensuring compliance with the Level III limit specified in groundwater guidelines (6.5–8.5) [27,28,29,30,31,32,33,34,35,36,37,38,39,40,41]. As a result, the mixture of red mud and loess is used to control the high alkalinity of the red mud, thereby enhancing its utility in engineering. The ordinary Portland cement used in the research had a strength grade of 42.5 and was sourced from the Taiyuan Lionhead Cement Plant.

Acidic mine wastewater containing Cd was simulated by dissolving the analytical reagent 3CdSO_4_∙8H_2_O in distilled water in a laboratory setting. The resulting heavy metal ion solution was precisely adjusted to the desired test concentrations and underwent pH modification using dilute sulfuric acid to mimic the acidity found in real acidic mine drainage. All chemical reagents were meticulously sourced from the Tianjin Chemical Factory, Tianjin, China, ensuring purity levels ranging between 98% and 101%.

### 2.2. Flexible Wall Penetration Test

In this test, a constant water head infiltration was employed, using equipment with a fixed water head to determine the permeability coefficient of a mixed material, including red mud and loess. The apparatus comprised a custom-designed dirt column segmented based on the desired filler height.

(1) Sample Preparation: A cylindrical mold, 5 cm tall and 5 cm wide, was cleaned, dried, and coated with Vaseline.
(2) Saturation: Using a peristaltic pump, distilled water was progressively transferred into the soil column in a bottom-up arrangement until the liquid level exceeded the sample by 3 cm. This process indicates specimen saturation.
(3) Measurement: The GeotestTK2000 flexible wall permeameter was employed following ASTM D5084-10 standards [42]. Osmotic pressure was 100 kPa, confining pressure was 110 kPa, and the hydraulic gradient was set to 200 for efficiency due to the sample’s firm strength and low permeability. The test involved vacuuming for 8 h at −100 kPa, introducing degassed water into the vacuum saturation cylinder, removing the saturated sample, placing it in the pressure chamber, injecting water, sealing it, and applying confining and osmotic pressure. After a three-day penetration test with hourly water production measurements, the instrument was turned off, and the sample was removed. The permeability coefficient is mathematically represented by Equation (1) based on test results.
  (1) $$k_{s}=\frac{Q}{iAt}$$
  where *k_s_* is the saturated sample permeability coefficient (cm/s); *i* is the seepage hydraulic gradient; *Q* is the cumulative seepage flow rate (cm^3^); *A* is the cross-sectional area of the seepage sample (cm^2^); and *t* is the time (s).

### 2.3. Shear Test

The shear test aimed to investigate the strength characteristics and hydraulic erosion resistance of the red mud–loess composite material under varying pore solutions. Specimens in the shear test underwent a preparation and testing procedure adhering fully to the geotechnical test method standard (GB/T 50123-2019) [43]. The quadruple strain gauge direct shear instrument was employed for the tests. After creating the red mud–loess mixed material using a layered compaction procedure in a ring cutter, the resulting sample was gradually inserted into the direct shear instrument. Direct shear tests were conducted on these samples, subjecting them to four vertical pressures: 100, 200, 300, and 400 kPa, involving various pore solutions. After applying vertical pressure, prompt removal of the fixed pin in the direct shear instrument was necessary. Subsequently, the sample underwent shearing at a rate of 0.8 mm/min, with real-time display of recorded values by the computer. If the curve exhibited stability or a notable recession, the maximum value of the curve was considered as the shear strength under vertical pressure. Conversely, if the curve continued to ascend, the shear strength under vertical pressure was determined by the corresponding shear stress at a shear deformation of 4 mm. The objective was to analyze the correlation between the four vertical pressures and the associated shear strength values, involving fitting the data to create a regression equation for shear strength, represented by Equation (2).

(2) τ=c+σtanφ

In the formula, τ is the shear strength of the red mud–loess mixed material, kPa; c is the cohesion of the red mud–loess mixed material, kPa; σ is the vertical pressure exerted on the red mud–loess mixed material during the shearing process, kPa; and φ is the internal friction angle of the red mud–loess mixed material.

### 2.4. Microscopic Testing and Characterization

#### 2.4.1. X-ray Diffraction Analysis (XRD)

X-ray diffraction (XRD) analysis is commonly employed for examining the phase composition of samples. The test sample, ground and sieved through a 0.075 mm sieve, underwent XRD scanning within a 2θ diffraction angle range of 10.00–60.00°. Specific operational parameters included a rotating target anode type (Cu), a test temperature of 22 °C, tube voltage and current set at 40 kV and 100 mA, respectively, with a scanning speed of 4°/min. Subsequently, the test results were analyzed using JADE 6.5 analysis software to determine the sample’s phase composition.

#### 2.4.2. Fourier Transform Infrared Spectroscopy (FTIR)

To prevent moisture from infiltrating the FTIR test sample, an appropriate amount of the test sample and KBr powder was combined in a mortar, illuminated by a drying lamp. The mixture was thoroughly mixed and ground evenly. Subsequently, the prepared sample was transferred into a mold and compressed into a translucent sheet using a tablet press. A pressure of 5–6 MPa was applied with a static pressing time of 1 min. Finally, FTIR scanning was conducted on the sample within a range of 400–4000 cm^−1^.

#### 2.4.3. Scanning Electron Microscope-Energy Dispersive X-ray Spectroscopy (SEM-EDS)

Commencing with a small ion sputtering instrument, evacuate the test sample and apply a gold spray to prevent charge accumulation. Subsequently, utilize the SEM to examine the surface micromorphology of the sample under 20 kV secondary electron conditions. Simultaneously, employ the EDS, integrated with JSM-IT200 (JEOL Ltd., Tokyo, Japan), to capture data from the corresponding SEM photos and analyze the elemental composition of the sample.

### 2.5. Methods

#### 2.5.1. Penetration Test

The loess used in this study originated from the topsoil layer. Loess samples with particle sizes above 0.5 mm contain various contaminants such as roots, leaves, stones, and similar materials. Therefore, fine-grained loess with particles smaller than 0.075 mm was chosen for this investigation. The fine-grained nature and high viscosity of red mud pose challenges to the permeability of the red mud–loess mixed material (7:3). To mitigate this, it is recommended to explore red mud with varying particle sizes and fine-grained loess for the mixing process.

This research aims to assess how variations in the particle size of red mud particles affect the mass ratio of red mud and loess. Before conducting the permeability test, the efficacy of mixtures of red mud and fine-grained loess with different particle sizes was evaluated. The focus was on their capacity to treat acidic mine wastewater containing Cd, with an initial pH value of 3.0. Five different mass ratios of red mud and loess were considered in this study. The test plan is detailed in Table 2.

**Figure 1:**
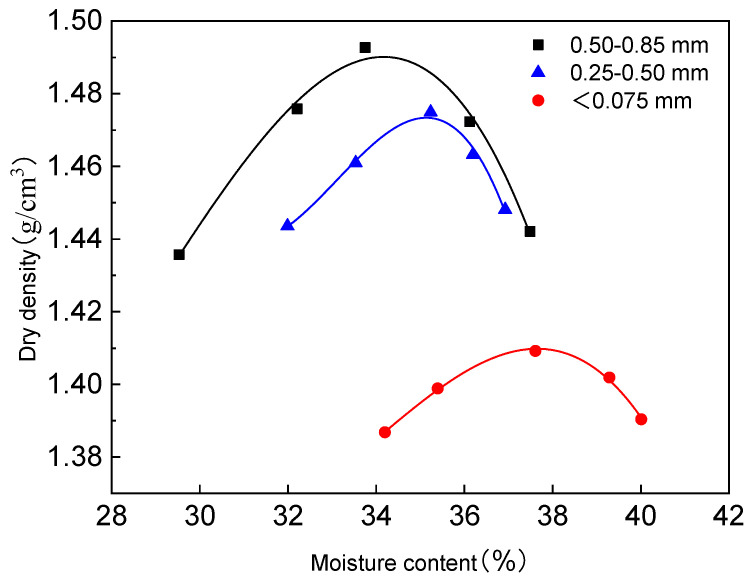
Compaction curves of red mud–loess mixture (7:3) under different particle sizes.

Light compaction tests were conducted on the red mud–loess mixed materials (7:3) with varying particle sizes according to the “Geotechnical Test Standard” (GB/T 50123-2019) [43]. The results, illustrated in Figure 1, show that larger particle sizes correspond to a reduction in the optimal moisture level and an increase in the maximum dry density for the red mud–loess mixed material. Smaller red mud particle sizes enhance water absorption in the composite, promoting the repolymerization of tiny particles and the formation of more non-closed pores. Consequently, the dry density of the fine-grained red mud–loess mixed material decreases. The ideal moisture level for the fine-grained mixture is 37.77%, while for the coarse-grained mixture, it is 34.19%. The fine-grained red mud–loess mixed material exhibits a maximum dry density of 1.41 g/cm^3^, whereas the coarse-grained counterpart reaches 1.49 g/cm^3^.

Firstly, select quartz sand particles larger than 1.08 mm and red mud–loess mixed material (7:3) with particles smaller than 0.075 mm. Combine them in the appropriate ratio to achieve the desired quartz sand content for optimal engineered barrier permeability. Secondly, blend red mud with varying particle sizes and fine-grained loess. Choose an initial moisture level that is slightly dry but within the ideal range. Layer and compact the red mud–loess mixture in a 7:3 ratio to a compaction degree of 70%. Precisely regulate the density of each soil sample layer during compaction to ensure uniformity, while investigating the red mud particle size suitable for engineered barrier permeability performance. Conduct a detailed penetration test according to the strategy outlined in Table 3.

#### 2.5.2. Shear Test

The research delved into assessing the shear strength evolution of the designed barrier’s active medium during its initial service term, impacted by pore water replenishment to comply with adsorption and permeability standards. It investigated various solutions, including pure water and an acidic solution with an initial pH of 3.0 and a concentration of 100 mg/L. This study scrutinized the shear characteristics of a red mud–loess mixture (7:3 ratio) with an initial pH of 3.0 and an acidic pore solution containing Cd. Additionally, the research explored the expected adsorption capacity of specific ions when the red mud–loess mixture (7:3) proved inadequate. An acidic pore solution with elevated Cd concentrations was prepared based on the equivalent weight of the soil sample and blended with the red mud–loess material in a 7:3 ratio to evaluate the shear characteristics. Through shear testing, this study assessed the impact of a coarse-grained red mud and loess mixture (7:3 ratio) on acid mine wastewater treatment and determined the theoretical adsorption capacity. The detailed test strategies are outlined in Table 4.

The experiment involved using several solutions as pore solutions. These included distilled water with an initial pH of 3.0, acidic solutions containing Cd with an initial concentration of 100 mg/L and an initial pH of 3.0, and four solutions with a total target ion content of 6 mg/g and an initial pH of 3.0. These solutions were mixed with the red mud–loess mixed material at an initial water content of 28%. Subsequently, the specimens were made using a ring knife with dimensions of Q × H = 61.8 mm × 20 mm and a dry density of 1.04 g/cm^3^. After being sealed for 72 h, shear tests were conducted. The precise configuration of the shear test is shown in Table 5.

#### 2.5.3. Microscopic Testing and Characterization

This study discusses the use of XRD, FTIR, and SEM-EDS for characterizing and analyzing the phase composition, functional groups, and elemental composition of samples, which includes measuring cadmium concentration in the red mud–loess mixtures used for acidic mine wastewater purification. These methods help elucidate the remediation mechanisms and assess the adsorption of cadmium (Cd) by the mixtures.

These techniques collectively contribute to measuring and understanding the concentration and behavior of cadmium in the red mud–loess mixtures.

## 3. Results and Discussion

### 3.1. Permeability Characteristics of Red Mud–Loess Engineered Barrier

#### 3.1.1. Effect of Quartz Sand Content

**Figure 2:**
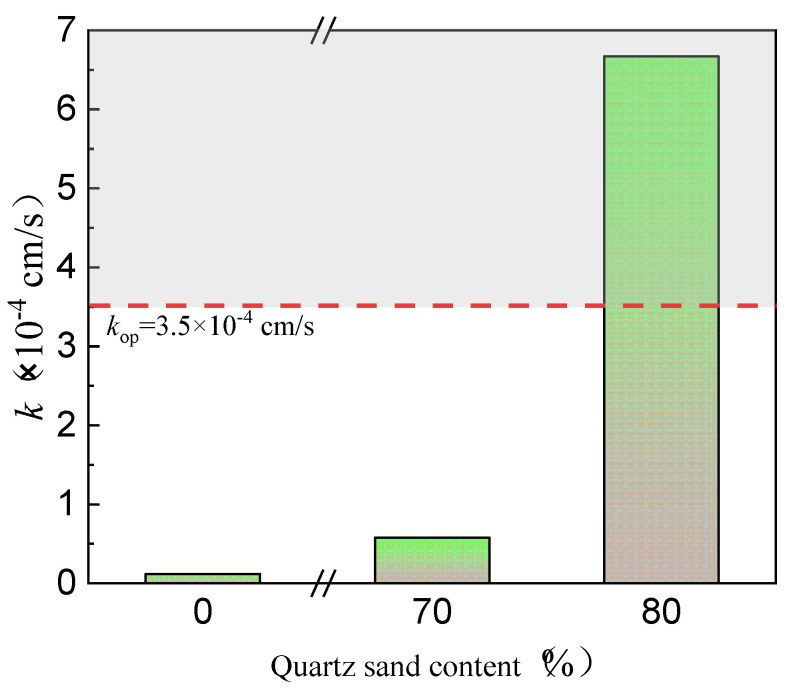
Permeability coefficient variations in solid waste with quartz sand content.

In Figure 2, the impact of quartz sand concentration on the permeability coefficient of the red mud loess mixtures is remarkable. A fine-grained red mud–loess mixture (7:3) with particles under 0.075 mm exhibits a poor permeability coefficient of 0.18 × 10^−4^ cm/s due to excessive viscosity in water. Quartz sand improves the permeability of red mud–loess mixed material (7:3). Exceeding an 80% quartz sand concentration significantly enhances permeability, reaching 6.7 × 10^−4^ cm/s. According to Zhang et al., 2019 [44], mixing powdered clinoptilolite with 95% quartz sand results in a permeability value of 6.32 × 10^−4^ cm/s. However, with 90% quartz sand, the decrease is sixfold.

Figure 2 shows that only quartz sand concentrations over 80% increase the permeability of the fine-grained red mud–loess mixed material (7:3). The red mud–loess constructed barrier used to remediate acid mine wastewater-polluted groundwater has 20% or fewer active components. This constraint reduces the service life of the designed barrier, requiring frequent replacement of wall media and leading to higher maintenance and monitoring expenditures. The use of quartz sand to increase the permeability of the red mud–loess constructed barrier is limited and needs improvement.

**Figure 3:**
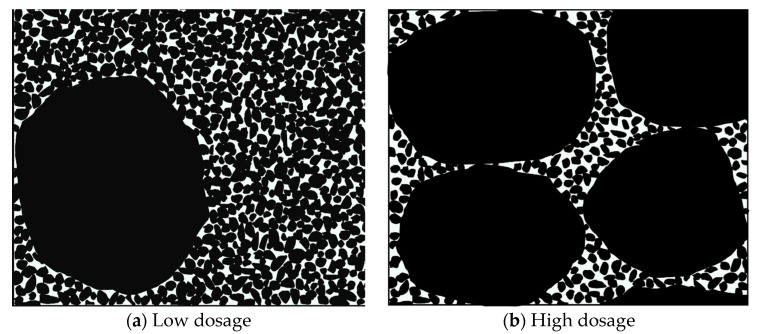
Particle arrangement in red mud–loess mix with varied quartz sand content.

Figure 3 illustrates particle stacking and organization in a red mud and loess (7:3 ratio) combination with varying quartz sand compositions. The research reveals that the composite material, comprising a 7:3 mix of fine-grained red mud and loess, contains more fine particles. This results in a dense and robust structure formed by completely filling interstitial spaces between particle frameworks. Consequently, this leads to inferior permeability performance, rendering improvement challenging, according to Hu et al., 2019 [45]; Ferreira et al., 2023 [46]; Wang et al., 2022 [47]; and Jang et al., 2023 [48].

Substantial quantities of the fine-grained red mud–loess mixed material (7:3) fill the gaps between quartz sand or larger particles at lower quartz sand concentrations. When combined with quartz sand and the red mud–loess mixture (7:3), the viscosity of red mud creates a complex network of flowable channels, reducing permeability. However, increasing the quartz sand concentration significantly diminishes the presence of the red mud–loess mixed material (7:3). This reduced presence of finely textured red mud–loess mixed material (7:3) allows quartz sand particles to adhere, thereby expanding the flow channel and enhancing permeability. 

#### 3.1.2. Particle Size Impact

(1) Optimal Mass Ratio for Coarse-Grained Red Mud–Loess Mixtures

**Figure 4:**
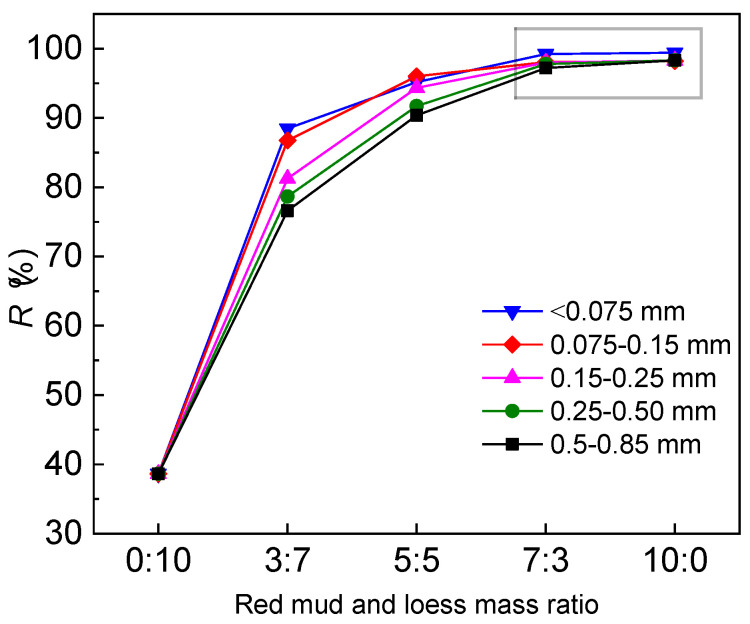
Impact of red mud to loess ratio on acid mine drainage treatment with varying particle sizes and initial Cd concentration.

The permeability test assessed a coarse-grained blend of red mud and loess to understand how particle size influenced the mass ratio. A mass ratio of 7:3 (red mud to loess) proved efficient in Cd removal from wastewater, while also increasing the acidity of the acidic wastewater to meet groundwater guidelines. Loess exhibits a strong ability to neutralize acidic solutions, making it particularly effective in removing heavy metals from wastewater (Wyszkowska et al., 2023 [49]). Figure 4 compares the mass ratio of red mud to loess with the treatment efficacy of mixed materials on acidic mine wastewater across various particle sizes. With an increase in the mass ratio of red mud to fine-grained loess, Cd removal rates from a mixture of five particle sizes of red mud and loess also increased. The red mud–loess mixture steadily removes target ions as the mass ratio approaches 7:3. Acidic compounds in the acid mine effluent may react with calcite-containing loess, hindering H^+^ ion accumulation in the red mud–loess mixture’s adsorption sites and active components. At a mass ratio of 7:3, red mud–loess effectively removes Cd in a similar way to pure red mud. Subsequent permeability studies have indicated that the optimal mass ratio of coarse red mud and fine-grained loess with varying particle sizes is 7:3. The ideal permeability and adsorption in an active barrier medium are inversely related. Larger red mud particles reduce the specific surface area of the red mud–loess mixture (7:3) and its active adsorption sites, decreasing Cd removal efficiency and enhancing the red mud and loess barrier permeability. Analyzing the red mud–loess mixture (7:3)’s adsorption capabilities and permeability coefficient is crucial for selecting the most suitable option (Wen et al., 2023 [50]).

(2) Permeability Characteristics of Coarse-Grained Red Mud–Loess Mixed Materials

**Figure 5:**
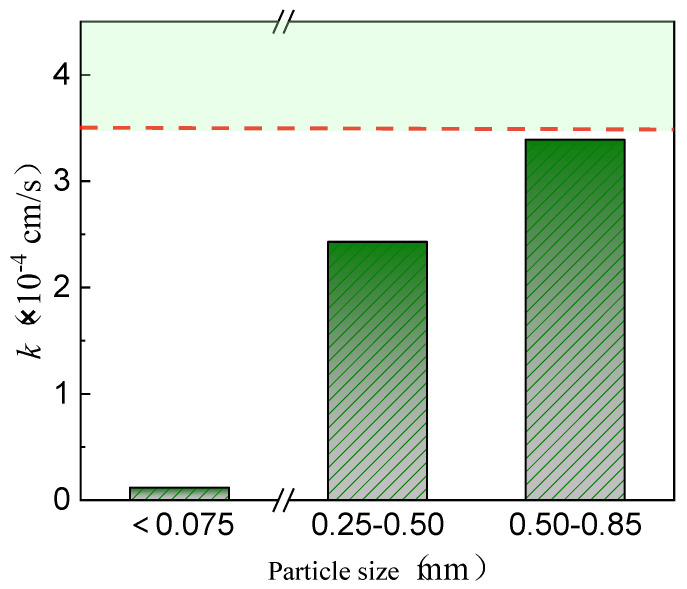
Permeability coefficient of red mud–loess mixture (7:3) with different particle sizes.

This study suggests that the quartz sand content significantly affects the permeability of fine-grained red mud–loess mixtures, with an optimal particle size range identified for maintaining effective permeability in engineered barriers. The mixture controls the high alkalinity of red mud, enhancing its utility in engineering applications such as barriers for acidic wastewater treatment (Wen et al. [50]). Figure 5 illustrates the permeability coefficient variation of a 7:3 mixture of red mud and loess with changing particle sizes. The data indicate that as the red mud particle size increases, the permeability coefficient of the red mud–loess mixture (7:3) progressively rises. This occurs because larger red mud particles leave interstitial spaces unfilled, enhancing permeability. Conversely, smaller particle sizes result in complete filling, reducing permeability due to stronger bonding. Mixtures of coarse-grained red mud and loess (7:3) with particle sizes below 0.50 mm exhibit permeability coefficients below 3.50 × 10^−4^ cm/s, while those in the 0.50–0.85 mm range hover around 3.39 × 10^−4^ cm/s, akin to 3.50 × 10^−4^ cm/s. Decreasing the dry density enhances the permeability. However, active components in engineered barriers degrade over time. For the effective treatment of acidic mine wastewater, utilize a 7:3 red mud and loess combination with particle sizes ranging between 0.50 and 0.85 mm. This selection ensures sustained permeability and reparability of the specified barrier, preventing pore blockage and mitigating decreases in the permeability coefficient due to adsorption and coagulation.

**Figure 6:**
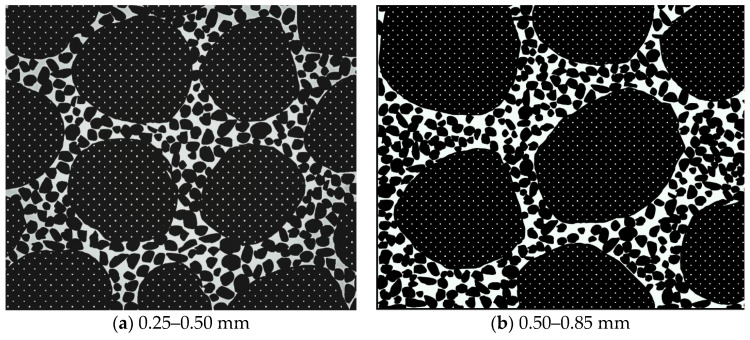
Particle arrangement in red mud–loess mix (7:3) with varied particle sizes.

Figure 6 illustrates the particle stacking and arrangement of the red mud–loess mixed material (7:3) across various sizes. Initially, dense arrangements of larger particles become looser as smaller particles fill the gaps between them. Conversely, placing large particles within a tightly packed system of smaller particles results in smaller particles adhering to the larger ones, creating a wall effect that increases space between particles and decreases density.

As depicted in Figure 6, when the particle size of the red mud–loess mixed material (7:3) decreases, the “coarse particles” acting as the skeleton diminish, while the number of smaller skeletons increases. This reduction in particle size diminishes the overall permeability pore channel, thereby reducing effective water seepage area and the permeability coefficient (Yu et al., 2019 [51]). Furthermore, increasing the red mud particle size decreases viscosity and enhances the skeleton effect of the red mud–loess combination (7:3), thereby improving permeability. The addition of quartz sand to the red mud–loess mixed material (7:3) increases particle size, subsequently enhancing permeability and concentrating active components in the designed barrier for treating acidic mine wastewater. This study investigates the use of red mud–loess mixtures for adsorbing cadmium from acidic mine wastewater, highlighting the importance of quartz sand content in enhancing permeability.

### 3.2. Shear Strength Properties of Red Mud–Loess Engineered Barrier

#### 3.2.1. Shear Stress Behavior of Red Mud–Loess (7:3) in Different Pore Conditions

(1) The Theoretical Adsorption Capacity of Coarse-Grained Red Mud–Loess Mixed Material (7:3):

Exploring the shear characteristics during the breakdown of coarse-grained red mud–loess mixed material (7:3) is crucial. However, the batch adsorption test did not align with the predicted adsorption capacity for specified ions when using the red mud–loess mixed material in a 7:3 ratio.

**Figure 7:**
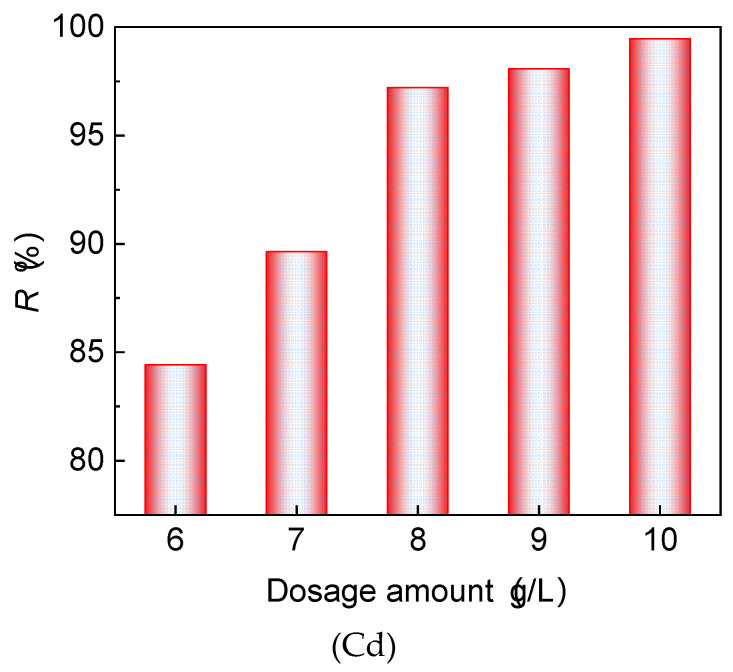
Treatment effect of Cd concentration dosage on acid mine wastewater.

Figure 7 depicts the relationship between the dosage of red mud–loess mixed material (7:3) and its efficacy in treating acid mine wastewater. Initially, the clearance rates of Cd show an upward trend, stabilizing as the quantity of red mud–loess mixed material (7:3) increases. Beyond Cd concentrations of 8 g/L, the mixture achieves a Cd removal effectiveness exceeding 97.5%. The optimal Cd concentration in the red mud–loess mixture (7:3) is identified as 8 g/L. Additionally, the red mud–loess mixed material (7:3) demonstrates theoretical adsorption capabilities of 13.5 mg/g for Cd. The shear testing reveals a theoretical adsorption capacity of 8 mg/g for target ions in the red mud–loess mixed material (7:3), derived from the theoretical adsorption capacity of Cd.

(2) Shear stress–shear displacement curve of red mud–loess mixed material (7:3)

**Figure 8:**
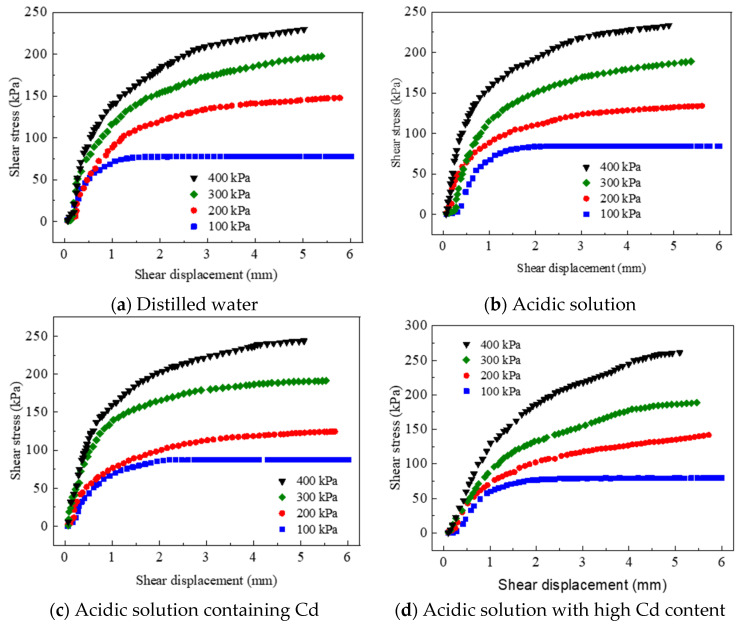
Shear stress displacement curve of solid waste in varied pore solutions.

Figure 8 illustrates the response of the red mud–loess mixed material (7:3) to various pore solutions, including pure water, an acidic solution with an initial pH of 3.0, and acidic solutions with an initial concentration of 100 mg/L of Cd and a starting pH of 3.0. This study examined four distinct conditions, maintaining a cumulative target ion concentration of 8 mg/g and an initial pH of 3.0, using acidic solutions containing Cd. Shear stress–shear displacement curves were recorded at vertical pressures of 100, 200, 300, and 400 kPa.

Analysis of Figure 8 reveals an initial abrupt spike in shear stress followed by a gradual leveling off as shear displacement increases. At a vertical pressure of 100 kPa, the material experiences its highest shear stress level and remains stable across all pore solutions. With increasing vertical pressure from 100 to 400 kPa, shear stress shows a consistent gradual rise. However, beyond 100 kPa, and across all four pore solutions, the material does not exhibit significant peaks in shear stress. This trend suggests a relationship of strain hardening between shear stress and shear displacement across the four pore solution settings. During early shearing stages, vertical pressure compresses pores between particles, increasing particle density and leading to a sudden shear stress rise. Higher vertical pressures expel air and water from the pores, densifying the structure and requiring increased shear stress for specimen collapse.

#### 3.2.2. Changes in Shear Strength Parameters

The shear strength of the red mud–loess mixed material (7:3) is governed by the combined frictional resistance and cohesion among its particles. The frictional resistance of the red mud–loess mixed material (7:3) exhibits a direct proportionality to the normal stress exerted on the sample. Nevertheless, the usual stress does not impact the cohesiveness of the material. Cohesion is determined by elements such as particle cementation and electrostatic attraction, leading to the existence of two distinct forms of cohesion: “original cohesion” which arises from electrical molecular interactions between particles, and “solidified cohesion” which results from cementation between particles.

**Figure 9:**
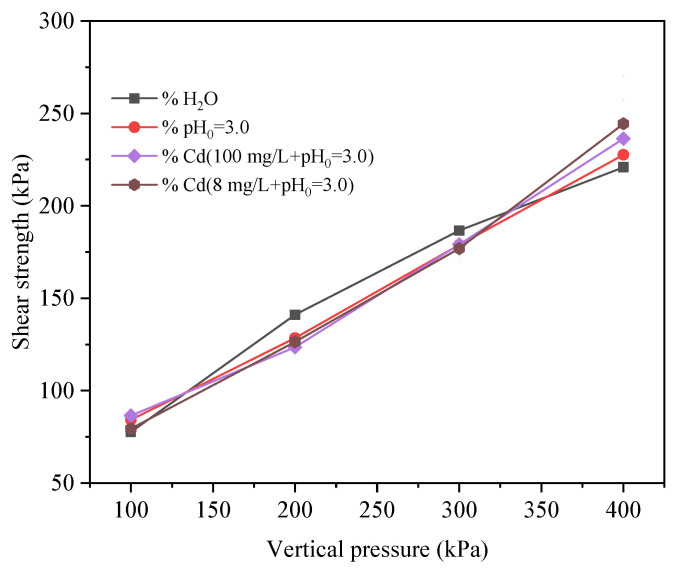
Shear strength vs. vertical pressure curve for solid waste.

By establishing the linear correlation between the shear strength and vertical pressure of the red mud–loess mixed material (7:3), we can determine the internal friction angle and cohesiveness of the red mud–loess mixed material (7:3) under four different pore solutions. Figure 9 depicts the correlation between shear strength and vertical pressure of a mixture of coarse-grained red mud and loess (at a ratio of 7:3) in the presence of four different pore solutions. The shear strength parameters acquired via the process of fitting are shown in Table 6.

The cohesion of the red mud–loess mixed material (7:3) with distilled water as the pore solution measures 37.80 kPa, with an internal friction angle of 25.42°, as detailed in Table 6. With increasing vertical stress from 100 kPa to 400 kPa, the shear strength gradually rises from 77.7 kPa to 220.9 kPa. For an acidic pore solution, the material displays a cohesion of 34.75 kPa and an internal friction angle of 25.65°, with shear strength increasing from 84.3 kPa to 227.6 kPa over the same vertical pressure range.

In the presence of a Cd acidic solution, the material exhibits a cohesion of 28.05 kPa and an internal friction angle of 27.37°, with shear strength increasing from 85.9 kPa to 235.7 kPa with increasing vertical pressure. Moreover, when subjected to an acidic solution with high Cd content as the pore solution, the material shows a cohesion of 20.55 kPa and an internal friction angle of 28.59°, with shear strength rising from 79.5 kPa to 244.4 kPa with increasing vertical pressure (Khalaf et al., 2023 [52]). Shear tests with various pore solutions provide insights into the shear strength parameters of the mixtures, validating solidification methods for contaminated soils (Nizioł et al., 2023 [53]).

The data in Table 6 provide valuable insights into the behavior of the red mud–loess mixed material (7:3) under varying conditions, highlighting distinct cohesion values and internal friction angles for each solution. The shear strength consistently increases with the rising vertical pressure for all solutions.

### 3.3. Research on the Environmental Safety Characteristics of Solidified Soil

#### 3.3.1. Acidity and Alkalinity Study in Cement-Solidification Cadmium-Contaminated Soil with Solid Waste

**Figure 10:**
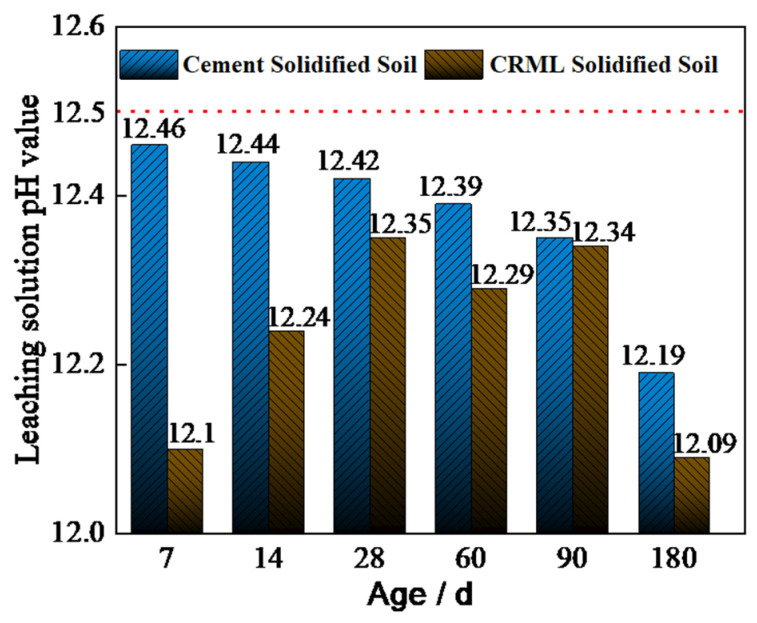
Relationship between pH and age of different kinds of solidified soils.

Figure 10 illustrates the pH variations in leachate from cement-solidified cadmium-contaminated soil over time, compared to cement–red mud–loess (CRML)-solidified soil. The pH of leachate from cement-solidified cadmium-contaminated soil gradually decreases. After 7 days, the pH of the cement-solidified leachate differs by 0.36 from the CRML-solidified leachate. Initially, the cement-solidified cadmium-contaminated soil has a higher pH than the CRML-solidified soil. Between days 7 and 28, the pH of the cement-solidified soil leachate drops by 0.04. Subsequently, from days 28 to 90, the pH reduces by 0.07, indicating a minor decrease before 90 days. From days 90 to 180, the pH decreases from 12.35 to 12.19.

At day 7, the leachate from the cement-solidified cadmium-contaminated soil has a peak pH of 12.46, nearing the minimum pH of 12.5 required by the “Hazardous Waste Identification Standard-Corrosive Identification” for hazardous waste leachate. Leachate from cement-treated cadmium-contaminated soil maintains a pH above 12.4 after 7–28 days, suggesting a significant concern. Figure 10 highlights that the CRML-solidified cadmium-contaminated soil exhibits lower pH values than the cement-solidified soil at various stages, indicating improved environmental safety.

#### 3.3.2. Cadmium Leaching Toxicity and Speciation in Cement-Solidified Soil with Solid Waste

To assess the leaching toxicity of CRML-solidified cadmium-contaminated soil over different durations (7, 14, 28, 60, 90, and 180 days), shear tests were conducted. Remarkably, no cadmium (Cd) was detected in the leachate, prompting the need for a detailed analysis of heavy metal content using the Tessier five-step extraction method. Distinct forms of cadmium entail diverse compounds in soil, providing insights into the bioavailability and mobility of cadmium, thereby deeply reflecting its toxicity in solidified soil. The Tessier five-step extraction method categorizes heavy metal forms into five as follows: exchangeable state, carbonate-bound state, iron and manganese oxide-bound state, organic matter-bound state, and residue state.

**Figure 11:**
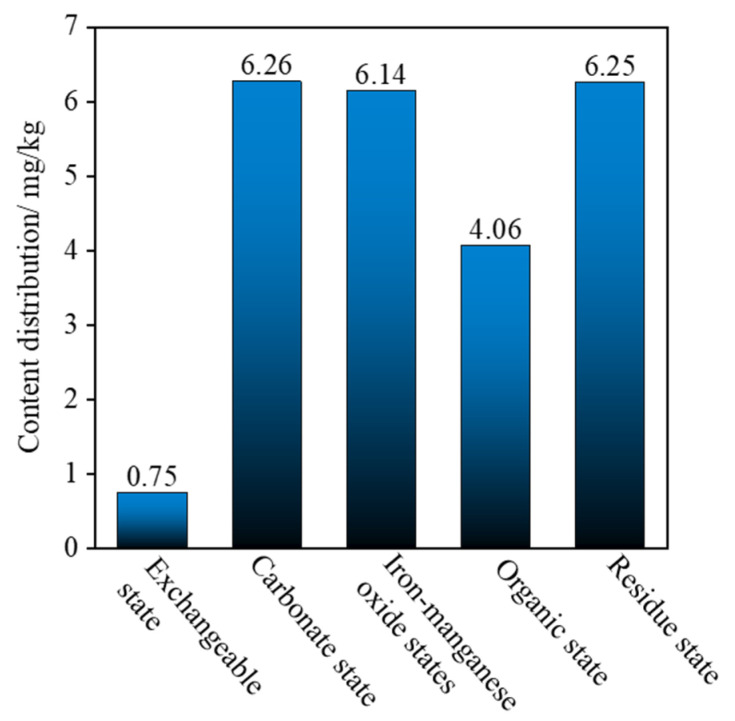
The distribution of cadmium content in different forms.

In a subsequent examination of 28-day-aged CRML-solidified cadmium-contaminated soil, Figure 11 illustrates the distribution of cadmium content across various forms. The total cadmium content in different forms was determined to be 23.45 mg/kg, closely aligning with the targeted Cd concentration of 25 mg/kg in artificially simulated contaminated soil. Noteworthy proportions in different forms include 3.19% in the exchangeable state, 26.69% in the carbonate-bound state, 26.19% in the iron and manganese oxide-bound state, 17.3% in the organic matter-bound state, and 26.64% in the residue state (Chen et al., 2021 [54]). Significantly, stable forms constitute 70%, while effective forms make up 30%, indicating the superior stabilizing effect of the cement and multi-source solid waste curing agent on cadmium. The research emphasizes the engineering characteristics of red mud–loess mixtures as active media for purification, offering valuable insights for environmental protection and sustainable development.

### 3.4. Microscopic Analysis of Stabilization and Solidification (S/S) of High-Concentration Heavy Metal-Contaminated Soil

#### 3.4.1. Comparison of Microscopic Morphology in Unsolidified and Solidified Cd-Contaminated Soil

**Figure 12:**
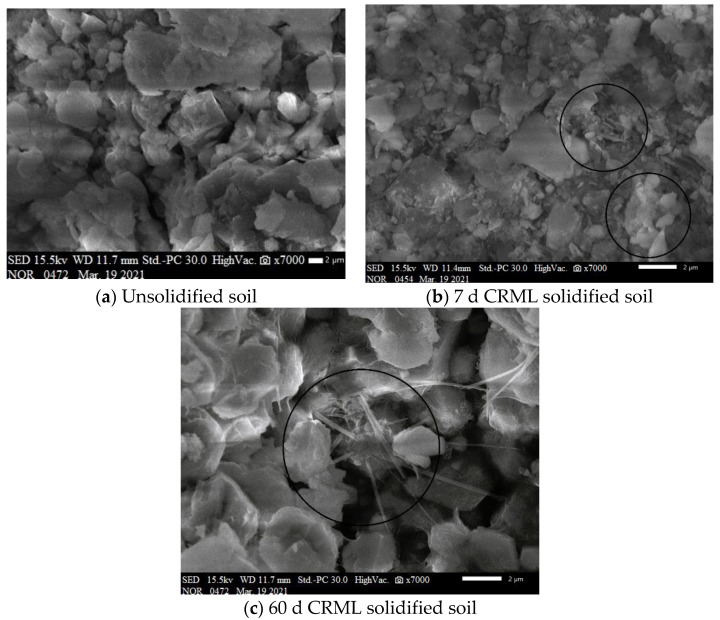
Microscopic morphology of contaminated soil and solidified soil.

SEM analysis was conducted on unsolidified and CRML-solidified samples at 7 and 60 days to determine pore conditions of cadmium-contaminated soil solidified with cement and multi-source solid waste (Figure 12). Unconsolidated Cadmium-Contaminated Soil (Figure 12a): SEM shows inconsistent blocks and granules. Particle adsorption alone does not connect soil particles without a curing agent. Stacking and extruding soil particles creates irregular hole patterns, resulting in a lack of a full skeletal structure and a generally closed pore region. Micromorphology shows needle-rod-shaped crystal products and flocculent gelling products in CRML-solidified soil at 7 days (Figure 12b). These overlapping, sturdy skeletons support soil particles, making them harder and more stable. Cemented soil particles and unhydrated solid waste are protected against uneven expansion and breaking by the gel-like hydration product. Gelled aggregates densify soil particles, clogging pores and decreasing penetration. CRML-Solidified Soil after 60 Days (Figure 12c): Needle-rod-shaped crystal products increase, supporting solid waste particles at pore openings. Density increases hydration product production, enhancing solidified soil structure and compactness. Comparing photos shows needle-shaped crystals gradually forming a structural framework and denser soil structure. Increasing solidified soil strength and impermeability is linked to continual hydration product manufacture.

#### 3.4.2. Functional Group Comparison in Unsolidified and CRML-Solidified Cadmium-Contaminated Soil

**Figure 13:**
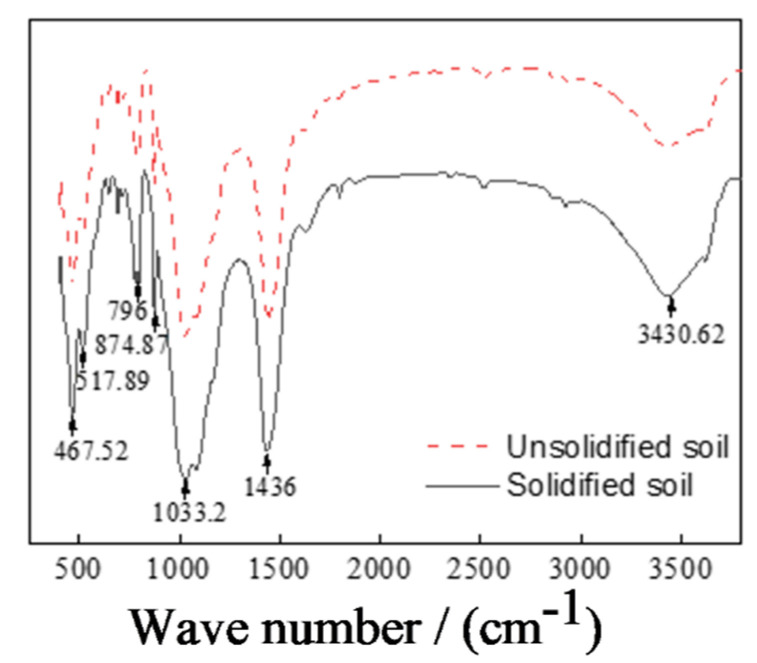
FTIR spectrum of uncured soil and solidified soil.

The CRML-solidified cadmium-contaminated soil undergoes significant changes in functional groups compared to unsolidified soil (Figure 13). Notable peaks include H_2_O stretching at 3430.62 cm^−1^, -OH stretching and bending at 1436 cm^−1^, and Al-O stretching at 517.89 cm^−1^, indicating the presence of adsorbed and crystallization water, AlO_6_^3−^ functional group, and sulfate ions. The presence of C-S-H gel, detected by Si-O asymmetric stretching at 874.87 cm^−1^ and in-plane bending at 467.52 cm^−1^, signifies hydration and calcium–silicate–hydrate formation. The Si-O peaks vary, with prominent peaks at 1033.2 cm^−1^ for asymmetric stretching, 796.07 cm^−1^ for symmetric stretching, and 467.52 cm^−1^ for symmetric angle-changing vibration. The addition of the curing agent enhances Si-O reactivity, generating amorphous silica and altering peak intensities. Vibrations of Si-O-Mg and Si-O-Al occur at 517.89 cm^−1^, while Si-O-Si vibrations are observed at 467.52 cm^−1^, consistent with XRD results. The -CO_3_^2−^ ion exhibits peaks at 1436 cm^−1^ and 874.87 cm^−1^, aiding in the formation of CaCO_3_ and CdCO_3_ during heavy metal contamination soil treatment.

#### 3.4.3. Mineral Composition Analysis of Solidified Cadmium-Contaminated Soil over Time

**Figure 14:**
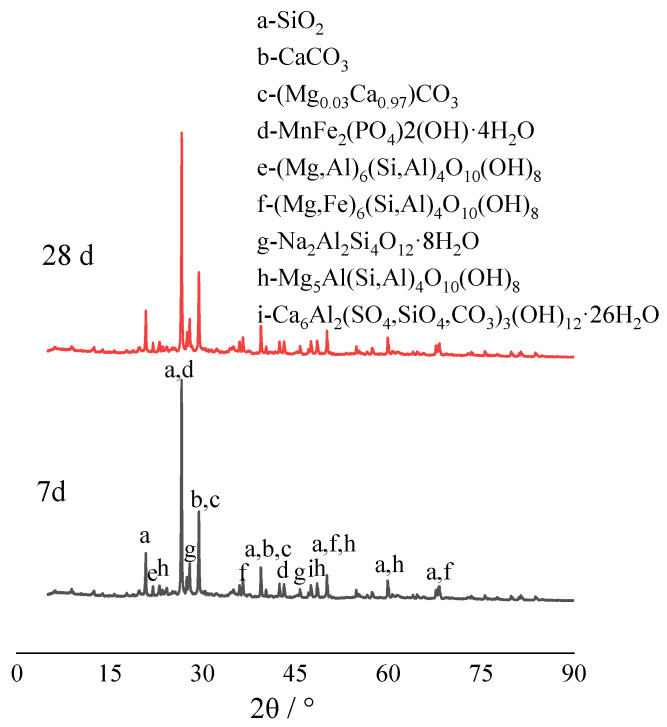
XRD of solidified soil.

X-ray diffraction (XRD) tests were performed on cadmium-polluted soil treated with calcium-rich by-product (CRML) after 7 and 28 days. The results, depicted in Figure 14, show that after 7 days, the phase composition of the CRML-treated cadmium-contaminated soil closely resembles that of the control block after 28 days, despite some differences between the solidified soil and test materials. SiO_2_, CaCO_3_, (Mg_0.03_, Ca_0.97_) CO_3_, MnFe_2_(PO_4_)_2_(OH)·4H_2_O, (Mg,Al)6(Si,Al)_4_O_10_(OH)_8_, and (Mg,Fe)_6_(Si,Al)_4_O_10_(OH)_8_ were identified in the solidified soil, alongside elements from the original materials. Compounds such as Na_2_Al_2_Si_4_O_12_·8H_2_O, Mg_5_Al(Si,Al)_4_O_10_(OH)_8_, and Ca_6_Al_2_(SO_4_,SiO_4_,CO_3_)_3_(OH)_12_·26H_2_O were also detected. The addition of curing agents, Ca_2_(SiO_4_) and Ca_3_(SiO_5_), may rapidly react with water, producing Ca(OH)_2_ and C-S-H, consistent with the gel-like hydration products observed in the SEM images. The presence of Ca(OH)_2_ from loess and cement may generate an extremely alkaline environment, enhancing the reactivity of red mud and clay minerals, thereby releasing active groups such as SiO_3_^2−^, SiO_4_^2−^, and AlO^2−^. Advanced analytical methods such as SEM-EDS, FTIR, and XRD are used to understand the processes involved in the remediation of soils contaminated with cadmium.

## 4. Conclusions

This research investigates Cd adsorption by red mud–loess mixed materials and evaluates the impact of quartz sand content on permeability. Shear tests using various pore solutions are conducted to analyze shear strength parameters. The study validates solidification methods for cadmium-contaminated soils and employs SEM-EDS, FTIR, and XRD analysis to reveal remediation mechanisms. Specific conclusions are as follows:

(1) Quartz sand amount influences fine-grained red mud–loess mixed material (7:3) permeability. A quartz sand content of less than 80% meets the permeability standards for red mud–loess barriers. The durability of the planned barrier diminishes when the composition of the red mud–loess mixture (7:3) deteriorates. Red mud and loess with particle sizes from 0.50 to 0.85 mm in a 7:3 mass ratio generate a 28% moisture-rich mixed material. With a dry density of 1.04 g/cm^3^, the red mud–loess mixture (7:3) satisfies the permeability standards for a barrier active medium, with a permeability coefficient of 3.39 × 10^−4^ cm/s.
(2) Loess has a strong ability to neutralize acidic solutions, which is particularly effective in removing heavy metals from wastewater. A mass ratio of 7:3 (red mud to loess) is found to be efficient in the removal of Cd, while also elevating the acidity of acidic wastewater to comply with groundwater guidelines. The mixture controls the high alkalinity of red mud, enhancing its utility in engineering applications such as barriers for acidic wastewater treatment. This study suggests that the quartz sand content significantly affects the permeability of the fine-grained red mud–loess mixtures, with an optimal particle size range identified for maintaining effective permeability in engineered barriers.
(3) Red mud–loess mixed materials (7:3) were tested in four pore solutions: distilled water, acidic solution, Cd-containing acidic solution, and high Cd-containing acidic solution. These materials initially exhibited an increase in behavior followed by gradual stabilization. Moreover, using a mass ratio of 7:3 for red mud to loess proved effective in removing Cd. The observed trend indicated strain hardening. Cd-loaded red mud–loess mixed material (7:3) demonstrated increased cohesiveness and a lower friction angle with higher target ion loading. However, as pollution increased, the cohesiveness of the Cd-loaded red mud–loess mixed materials decreased, despite an increase in the internal friction angle. Interestingly, after loading Cd, the red mud–loess mixed material (7:3) exhibited somewhat greater shear strength than uncontaminated samples.
(4) The red mud–loess mixed material includes Cd acidic solutions (7:3) and the pore solution is acidic. With distilled water as the pore solution and red mud–loess mixed material (7:3), H^+^ and target ions disrupt particle aggregation. The clay component in the red mud–loess mixed material (7:3) lacks cement because carbonates degrade. The red mud–loess mixed material (7:3) becomes more irregular and rough owing to H^+^ corrosion in the acidic solution and Cd adsorption products adhering.
(5) SEM analysis of the CRML-solidified soil at 7 and 60 days reveals irregular blocks and granules with needle-rod-shaped crystals, which enhance soil structure and strength over time. CRML-solidified cadmium-contaminated soil undergoes significant alterations in functional groups (-OH and Si-O), indicating the presence of adsorbed water, crystallization water, and mineral formations. The curing agent triggers reactions that enhance soil reactivity, leading to the formation of amorphous silica, calcium carbonate, and cadmium carbonate. XRD analysis of CRML-treated cadmium-contaminated soil demonstrates consistent phase composition between 7 and 28 days. Additionally, the curing agent facilitates the liberation of active groups, thereby boosting soil reactivity. These findings contribute to the understanding of soil remediation and the efficient utilization of solid waste resources in environmental protection. This study’s innovative approach using red mud and loess as PRB active media demonstrates its potential for cleansing acidic wastewater in mining areas.

## Figures and Tables

**Table 1:** The key chemical constituents of red mud and loess.

Composition	SiO_2_	Al_2_O_3_	Fe_2_O_3_	CaO	K_2_O	MgO	TiO_2_	Na_2_O	MnO	SO_3_
Red mud	20.17	24.34	9.40	18.26	0.64	1.26	3.56	9.61	0.03	0.47
Loess	58.88	11.75	4.54	7.98	2.18	2.05	0.60	1.70	0.07	0.03

**Table 2:** Adsorption parameters for Cd on red mud–loess mixtures with particle size variations.

Red Mud–Loess Mass Ratio	Particle Size (mm)	pH_0_	Dosage (g/L)	Contact Time (min)	Initial Concentration (mg/L)	Temperature (°C)
0:10	<0.075	3.0	8 (Cd)	600	100	25
3:7	0.075–0.15					
5:5	0.15–0.25					
7:3	0.25–0.50					
10:0	0.50–0.85					

**Table 3:** The operating conditions of permeability test.

Sample Size ϕ×H (cm)	Quartz Sand Content (%)	Particle Size (mm)	Initial Moisture Content (%)
5 × 10	0	<0.075	30
	70	<0.075	8
	80	<0.075	5
	0	0.25–0.50	29
	0	0.50–0.85	28

**Table 4:** Adsorption conditions for heavy metals on solid waste with varying dosages.

Red Mud–Loess Mass Ratio	Particle Size (mm)	pH_0_	Dosage (g/L) Cd	Contact Time (min)	Initial Concentration (mg/L)	Temperature (°C)
7:3	0.50–0.85	3.0	6	600	100	25
			7			
			8			
			9			
			10			

**Table 5:** The conditions of the shear test plan.

Sample No	Sample Size ϕ×H (mm)	Vertical Pressure (kPa)	Pore Water Category
1	61.8 × 20	100	Distilled water (H_2_O)
2		200	Acidic solution (pH_0_ = 3.0)
3		300	Acidic solution containing Cd *C*(Cd) = 100 mg/L, pH_0_ = 3.0
4		400	Acidic solution with high Cd content *m*(Cd) = 6 mg/g, pH_0_ = 3.0

Note: *m*(Cd) both refer to the ratio of the mass of heavy metal ions Cd in the sample to the mass of red mud–loess mixed material (7:3).

**Table 6:** Shear parameters of red mud–loess mixtures with different pore solutions (7:3).

Pore Solution	*c* (kPa)	*φ* (°)	tan *φ*	*R* ^2^
Distilled water (H_2_O)	37.80	25.42	0.4752	0.9719
Acidic solution (pH_0_ = 3.0)	34.75	25.65	0.4803	0.9989
Acidic solution containing Cd *C*(Cd) = 100 mg/L, pH_0_ = 3.0	28.05	27.37	0.5176	0.9735
Acidic solution with high Cd content *m*(Cd) = 6 mg/g, pH_0_ = 3.0	20.55	28.59	0.5451	0.9885

## Data Availability

Data presented in this study are available on request from the corresponding authors due to restrictions privacy.
